# Peer Review of “In-hospital Mortality and the Predictive Ability of the Modified Early Warning Score in Ghana: Single-Center, Retrospective Study”

**DOI:** 10.2196/30763

**Published:** 2021-07-12

**Authors:** Ana Marusic

**Affiliations:** 1 School of Medicine University of Split Split Croatia

**Keywords:** modified early warning score, MEWS, AVPU scale, Korle-Bu Teaching Hospital, KBTH, Ghana, critical care, vital signs, global health

*This is a peer-review report submitted for the paper “In-hospital Mortality and the Predictive Ability of the Modified Early Warning Score in Ghana: Single-Center, Retrospective Study.”*

## Round 1

### General Comments

This paper [1] presents a comparison of the limited modified early warning score (LMEWS) versus the standard MEWS in their ability to predict in-hospital mortality in Ghana. The authors demonstrate that LMEWS is a good predictor of in-hospital mortality, especially in lower-resource health care settings.

The study is well executed and well written, but I am not sure whether it aligns with the scope of *JMIRx Med*—this is a purely clinical study relevant to a public health or anesthesiology journal.

### Specific Comments

#### Major Comments

My main comments are about the methodology of the article:

1. There is no explanation on how the study size was arrived at.
2. It is not clearly described whether there any missing data and how they were handled.
3. It is not clear whether there was an attempt at a blind assessment of the predictors.
4. A flow chart of the patients in the study is absent, including the time of follow-up with patients.
5. The ethics considerations were not sufficiently addressed. What kind of approval was obtained for the retrospective secondary use of data? Minors (patients were aged 13 years and older) were also included so the question of assent is also relevant here.

## Round 2

### General Comments

This paper [1] presents an interesting observation on the predictive ability of the modified early warning score on in-hospital mortality among critically ill patients.

The revised manuscript addresses some of the concerns, but I am not sure that all of them are satisfactorily answered.

### Specific Comments

#### Major Comments

1. All reviewers expressed concerns about the sample size, and it is still not clear whether the sample size was calculated before the study. The response was that the sample size was calculated to be 82 participants, but it is not clear whether this was for the whole study (all 4 groups in the flow chart representing the flow of participants) or for individual groups. Also, there was a disbalance between the size of the 4 groups (81 with a nonsignificant MEWS and 31 with a significant MEWS, and 79 with a nonsignificant LMEWS and 33 with a significant LMEWS).
2. The question about missing data was addressed, and there was only a single case of missing data.
3. The blinding of the assessor was not performed. Although the authors argue that it was not necessary, it is an important methodological tool to address biases in analyses.
4. I am not satisfied with the response regarding the ethics approval of the study. It is not clear whether the patients or their parents consented to the inclusion of data collected during medical procedures in a research study.
